# Aberrant pancreatic tissue in a giant mediastinal cyst: an uncommon entity

**DOI:** 10.1186/s13019-019-0955-2

**Published:** 2019-07-08

**Authors:** Josiah Miner Njem, Benjamin Ugwu, John Collins, Innocent Emmanuel, Ojile Akpa Philip, John Awodi

**Affiliations:** 1 Department of Paediatrics, Jos University Teaching Hospital, Jos, Nigeria; 2 Department of Pathology & Morbid Anatomy, Jos University Teaching Hospital, Jos, Nigeria; 3 Cardiothoracic Surgery Unit, Department of Surgery Jos University Teaching Hospital, Jos, Nigeria

**Keywords:** Mediastinal cyst, Heterotopic pancreatic tissue

## Abstract

**Background:**

Heterotopia of pancreatic tissue in the thorax and mediastinum is uncommon, although, a common developmental anomaly in the gastrointestinal tract where the aberrant tissue is a component of gastrointestinal duplication cysts, intralobular pulmonary sequestration or teratomas.

**Case presentation:**

We report a case of an ectopic pancreas located in a giant mediastinal cyst in a 2-year old girl who presented with severe dyspnoea due to external compression of the intra-thoracic structures, mainly the right main bronchus. Surgical resection was carried out with complete relief of symptoms. The cyst was confirmed histopathologically to contain pancreatic tissue. Literature review showed that this is an uncommon presentation hence the need to report the case.

**Conclusion:**

Heterotopia of the pancreas although, an uncommon occurrence in the thorax and mediastinum, should be considered when dealing with cystic lesions of the mediastinum and surgery plays an important role in its management.

**Trial registration:**

JUTH/DCS/ADM/127/XXV/203. Registered 2nd September 2018.

## Introduction

Heterotopic pancreas, also known as aberrant or ectopic pancreas, is pancreatic tissue which has no anatomic and vascular continuity with the main body of the pancreas [1–5]. Congenital ectopia of pancreatic tissue although, a common developmental anomaly in the gastrointestinal tract, its occurrence in the thorax and mediastinum is uncommon [1–3]. ,Aberrant pancreatic tissue in the anterior mediastinum was first described pathologically by Klob in 1859 and first published by Shillitoe and Wilson in 1957 [4, 6, 8]. When found in the thorax, the pancreatic tissue is mostly a component of gastroenteric duplication cysts, intralobular pulmonary sequestration or teratomas [1, 4–8]. This anomaly has been reported in approximately 2% of autopsies and 70–90% of these were located in the gastrointestinal tract mainly in the stomach, duodenum, jejunum and ileum [1–3]. The aetiology of this anomaly is unclear, there are two theories regarding its embryogenesis [5, 6]. The first theory involves abnormal differentiation of the pluripotent epithelial cells of the ventral primary foregut resulting in the formation of ectopic pancreatic tissues in the mediastinum [5–8]. This was supported by the fact that the pancreas and lower respiratory tract, share the same embryological origin from the primitive foregut. The second theory involves migration of cells from the pancreatic bud to the different sites [6].

Clinical presentations are non-specific and essentially result from compression of intra-thoracic structures, thus, symptoms such as cough and dyspnoea due to compression of the major airway may occur. Hypoglycaemia has also been reported in patients with ectopic pancreatic tissue in the mediastinum [4]. We carried out Pub med search to find all the reported cases of ectopic, heterotopic or aberrant pancreatic tissue in the mediastinum and found that in addition to the index patient, there were thirty reported cases of aberrant pancreas in the mediastinum (Table 1). Most of the cases described were in young adults, the average age was 29 years. The index patient was a two year old girl. The prevalence was higher in females (58%). Aberrant pancreas in the mediastinum is uncommon and to the best of our knowledge thirty cases have been documented in the literature (Table 1). We report an uncommon clinical case of a giant mediastinal cyst containing aberrant pancreatic tissue.

**Table 1:** Ectopic pancreas in the anterior mediastinum- A report of 30 patients [6, 8]

SN	Reference	Gender	Age (years)	Size (cm)	Pathology
1	Shillitoe etal 1957	F	15	5.5	benign
2	Carr et al. 1977	F	57	10	benign
3	Von Schweinitz et al. 1990	M	5	5x5x5	benign
4	Perez-Ordonez et al. 1996	F	16	12	benign
5	Gong et al. 1997	F	26	20 × 15	benign
6	Gong et al. 1997	F	26	4.3 × 1.3	benign
7	Wu et al. 1998	F	60	10 × 15	benign
8	Cagirici et al. 2001	F	45	10 × 8	benign
9	Sentis et al. 2004	M	44	10x8x7.5	benign
10	Yamato et al. 2005	M	39	10 × 8	benign
11	Al-Salam et al. 2006	M	40	8x6x6	benign
12	Wang et al. 2007	M	17	12x12x4	benign
13	Wang et al. 2007	F	24	10x8x4	benign
14	Ehricht et al. 2009	M	25	15 × 15	benign
15	Chen et al. 2009	F	32	13x16x8	benign
16	Fayoumi et al. 2010	M	51	10x7x5	benign
17	Fayoumi et al. 2010	M	42	10 × 5	benign
18	Takemura et al. 2011	F	21	3.5 × 3.5	benign
19	Sandor et al. 2012	M	32	4 × 4	benign
20	Byun CS et al. 2012	F	31	7x3x4	benign
21	St Romain et al. 2012	F	66	11x	malignant
22	Rokach et al. 2013	F	22	5.1 × 3.8 × 2.3	benign
23	Zhang et al. 2014	M	15	7 × 4.5	benign
24	Zhang et al. 2014	F	16	6	benign
25	Li et al. 2014	M	18	16x12x9	benign
26	Sibel et al. 2014	M	23	6 × 8	benign
27	Koh et al. 2015	M	17	7.5x7x5.5	benign
28	Wu et al. 2015	F	45	7.5x7x5.5	benign
29	Mansi et al. 2017	F	29 days	5x4x3.5	benign
30	Snak et al	F	21	6.7 × 7.5	benign
31	Index case	F	2	20x16x3.5	benign

## Case report

**Fig. 1:**
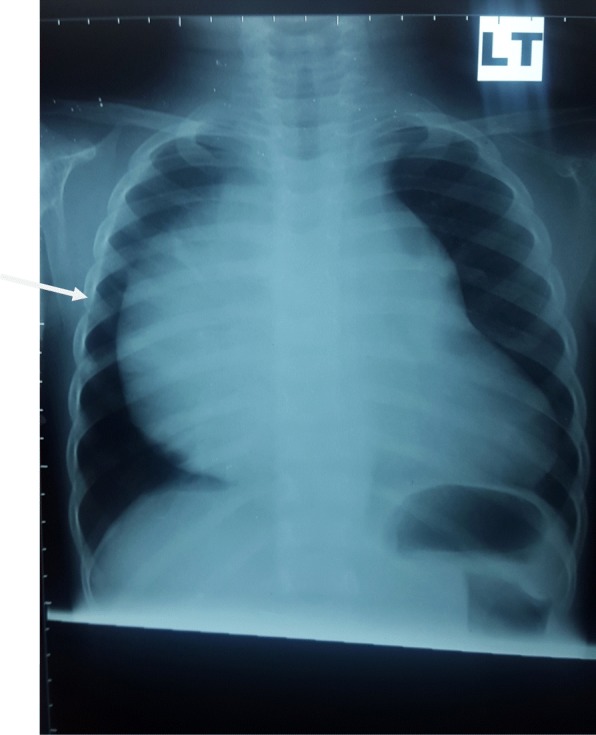
Chest X-ray showing widdened mediastinum(arrow) and homogenous opacity continous with the cardiac silhouette (arraw)

**Fig. 2:**
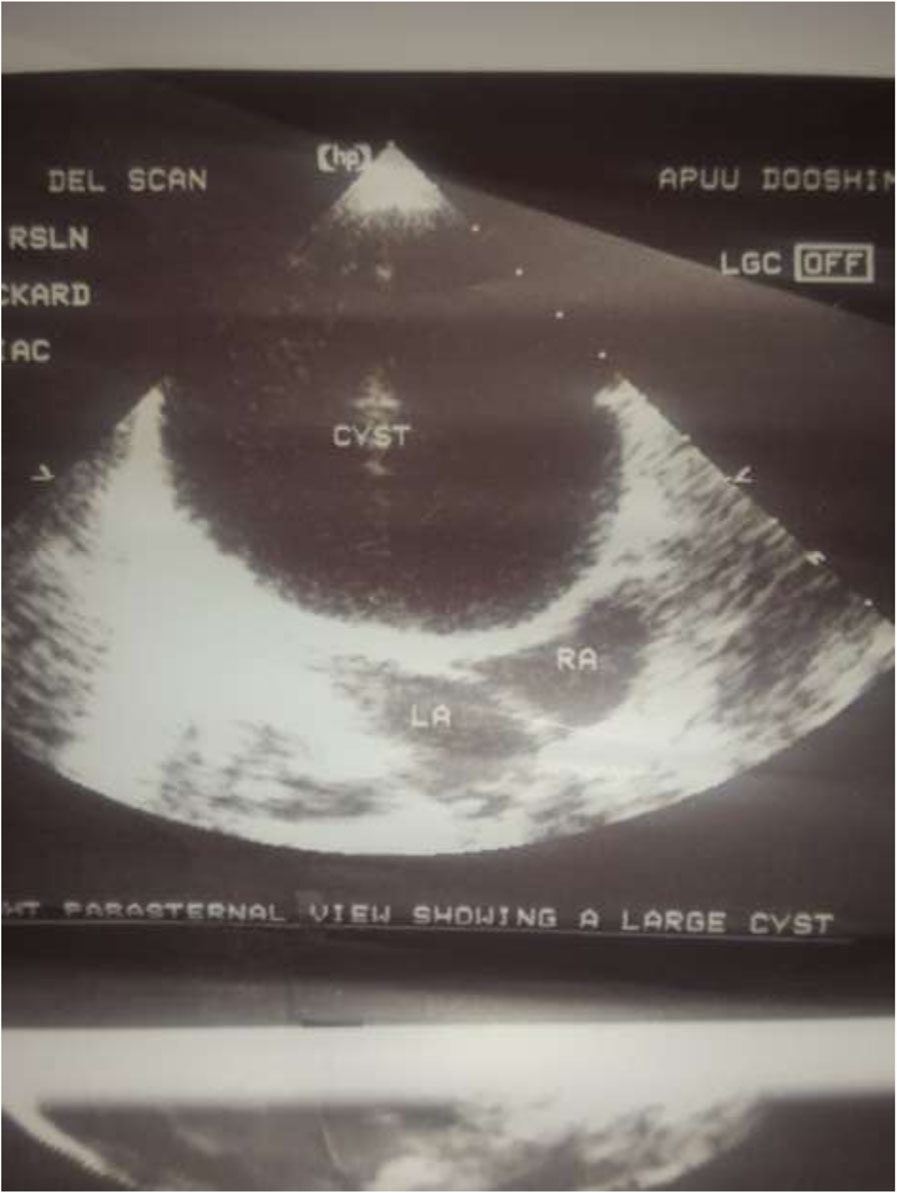
Echocardiography showing the cyst

**Fig. 3:**
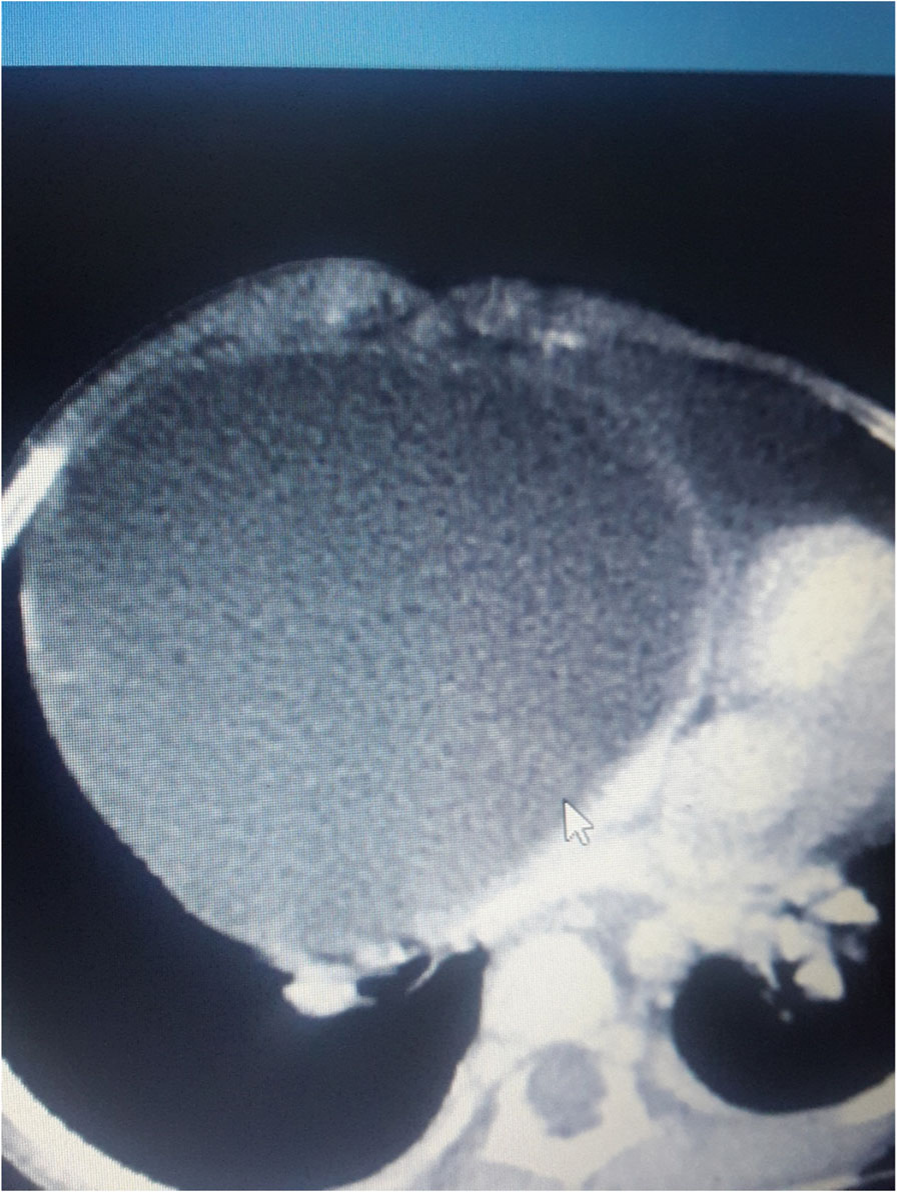
Contrast CT scan showing the cyst(white arrow)

**Fig. 4:**
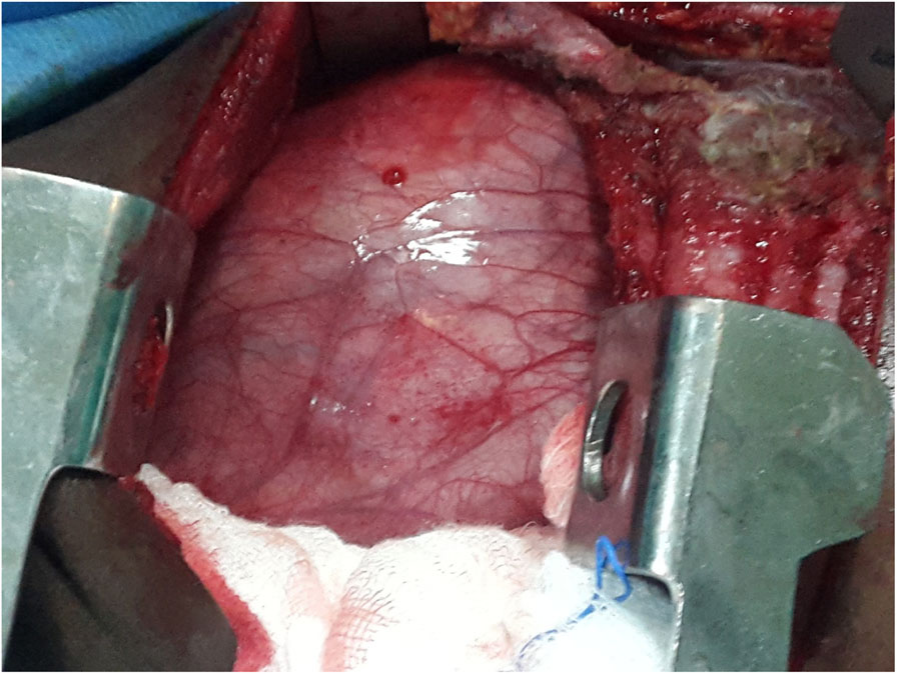
Intra-operative picture of the cyst(arrow)

**Fig. 5:**
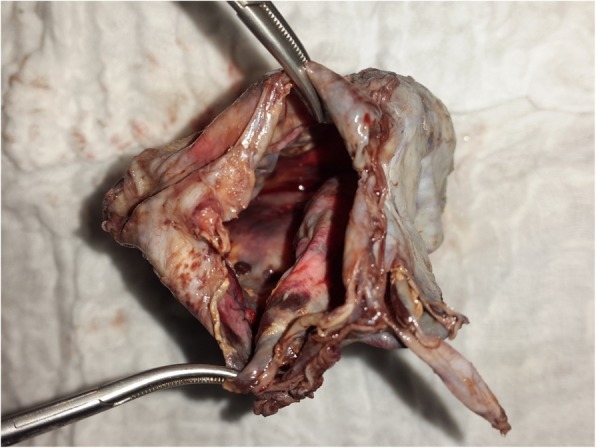
Cyst after immersion in formalin

**Fig. 6:**
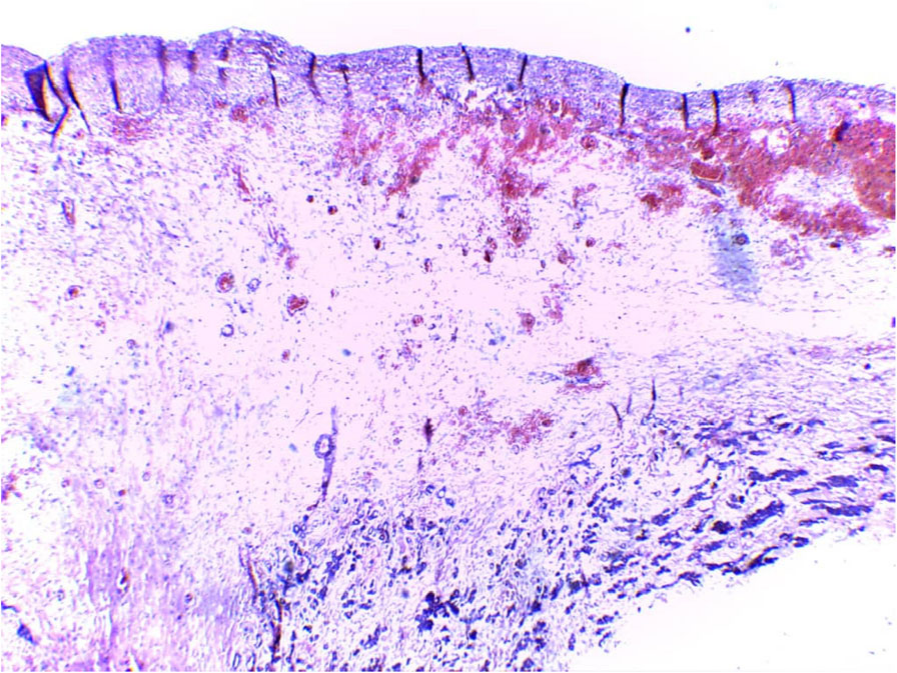
Photomicrograph shows cyst wall with fibrous capsule overlying a fibromyxoid stroma, within the stroma are seen congested blood vessels and numerous acini lined by cuboidal epithelium(H&E X14)

**Fig. 7:**
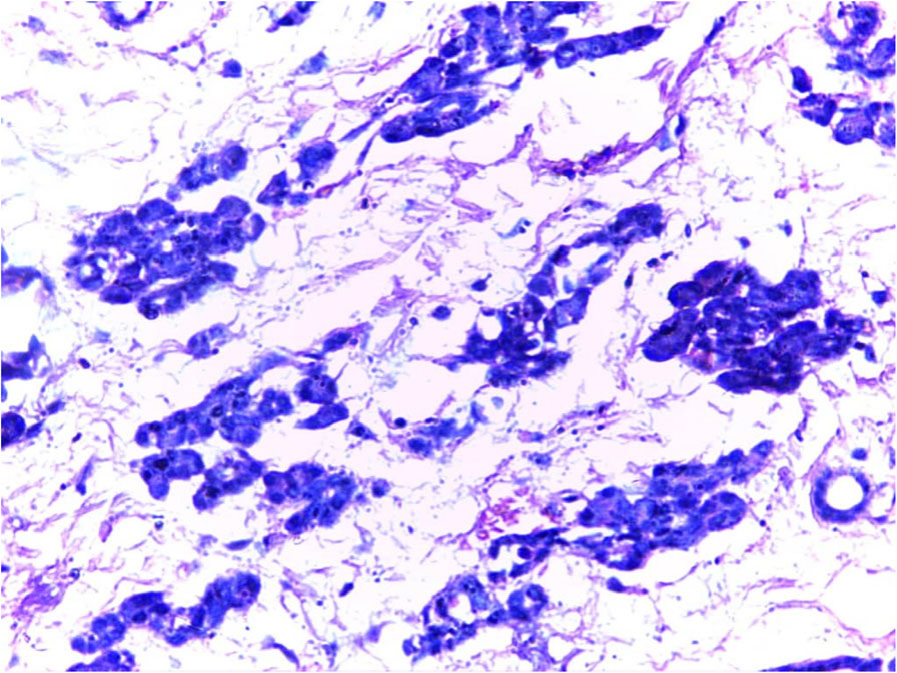
Acini lined by cuboidal epithelial cells with small round to oval nuclei with basophilic cytoplasm (H&E X10)

**Fig. 8:**
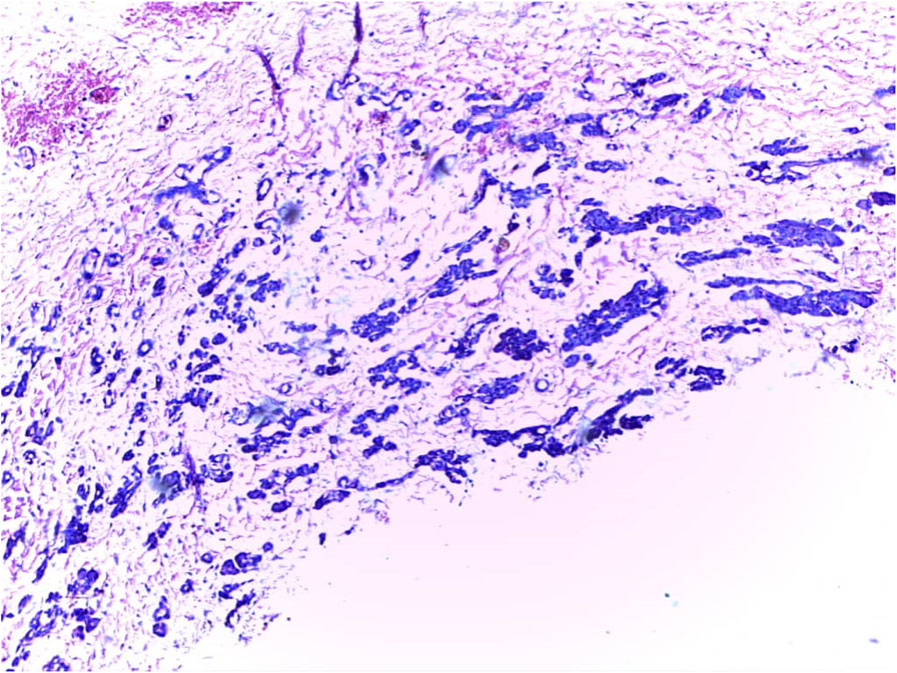
Acini with fibromyxoid stroma lined by cuboidal epithelium with small oval nuclei and basophilic cytoplasm (H&E X40)

A 2-year old girl was referred to the Jos University Teaching Hospital in north central Nigeria, with complains of progressive cough and difficulty in breathing in the preceding six months. She was treated at several health facilities for pneumonia and asthma without resolution of symptoms. Patient had no fever or features suggestive of tuberculosis. Pregnancy, delivery, neonatal and infant periods were uneventful and patient had received all immunization appropriate for her age. Examination at presentation showed a child in severe respiratory difficulty necessitating oxygen administration, with Spo2 89–90% at room air and 96% on oxygen by nasal prongs. There was bulging of the right side of her anterior chest wall and markedly reduced breath sounds on the right hemithorax. No features suggestive of congenital heart disease. Chest X-ray showed homogenous opacity continuous with the cardiac silhouette, involving almost the entire right thoracic cavity with a shift of the mediastinum to the left (Fig. 1). Echocardiography confirmed a very large well defined mediastinal cyst compressing the right atrium, left atrium and right ventricle (Fig. 2). Contrast-enhanced computed tomography scan of the chest showed a well defined large cyst of the anterior mediastinum compressing the right main bronchus (Fig. 3). Blood investigations were all normal. A diagnosis of anterior mediastinal cyst was made and the patient had right posterolateral thoracotomy through the fifth intercostals space. Intraoperatively a large tense cyst measuring 20 × 16 × 3.5 cm was noted adjacent to the pericardium and attached loosely to the thymus compressing but not attached to the bronchus. The cyst was unilocular with a thick wall and contained serous fluid (Figs. 4 & 5); the cyst was excised en bloc. There was moderate pericardial fluid which was drained by a pericardiostomy. Histopathological sections showed ectopic pancreatic tissues in the wall of the cyst (Figs. 6, 7 and 8). The post operative course was uneventful; the patient has been asymptomatic after a followed-up period of twenty four months.

## Discussion

Pancreatic heterotopia, also referred to as ectopic or aberrant pancreas is pancreatic tissue that has no anatomic or vascular connection with the main body of the pancreas [2–6]. This anomaly has been reported in approximately 2% of autopsies and 70–90% of these are located in the gastrointestinal tract mainly in the stomach, duodenum, jejunum and ileum [1–3]. Congenital ectopia of pancreatic tissue in the thorax and mediastinum is quite uncommon and occurs mostly in adults, with a slight female preponderance [1–12]. The index patient was a two old year girl.

The clinical presentation was usually non specific, related to the size and location of the lesion as well as the presence of inflammation or malignant transformation within the cyst. The patients usually present with features such as cough, shortness of breath, chest pain, fatigue, shoulder pain, fever, night sweats, and pulmonary infiltrates, pleural and pericardial effusion [3, 5–10]. The index patient presented with shortness of breath, cough, homogenous opacity on chest radiograph and pericardial effusion. We considered these symptoms to be due to compression of adjacent structures by the large cyst, since symptoms completely resolved following surgical excision of the cyst and patient has remained asymptomatic at follow-up.

Contrast-enhanced computed tomography scan and echocardiography were useful in the diagnosis of mediastinal cysts. There were however, no specific features on either of these imaging modalities to distinguish ectopic pancreatic tissue from other cystic lesions of the mediastinum. Most cases were benign and had complete resolution of symptoms after surgery, with no recurrence [6–12]. The index patient had complete resolution of symptoms and has remained asymptomatic at follow-up. The histopathology showed benign cystic lesion containing ectopic pancreas. This underscores the importance of surgery in the management of this lesion, which could prevent malignant changes as well as relieving pressure on adjacent structures.

## Conclusion

Pancreatic heterotopia should be considered in the differential diagnosis of mediastinal cyst. Most mediastinal cyst containing ectopic pancreatic tissues are benign and surgical resection results in complete resolution of symptoms, thus strengthening the importance of surgery in the management of this lesion.

## Data Availability

not applicable.
